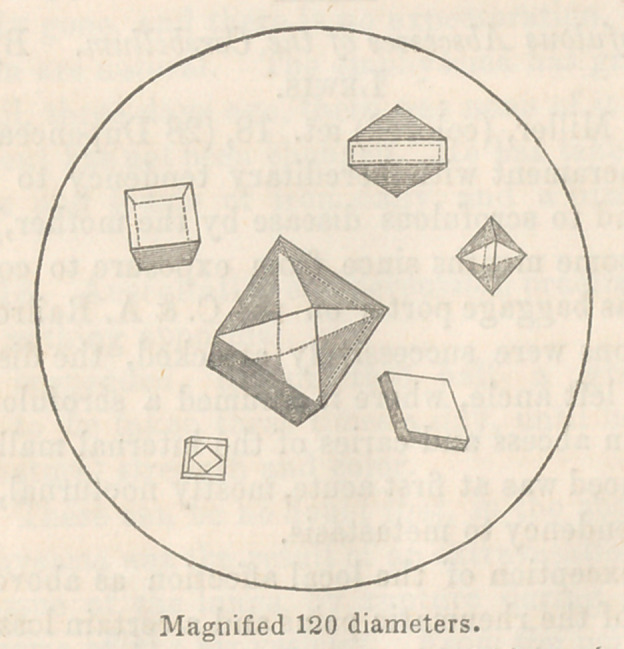# Case of Scrofulous Abscesses of the Cerebellum

**Published:** 1854-01

**Authors:** F. W. Lewis


*Case of Scrofulous Abscesses of the Cerebellum.* By Dr. F. W. Lewis.

Alexander Miller, (colored) æt. 18, (28 Duponceau street,) of nervous temperament with hereditary tendency to phthisis (by the father, and to scrofulous disease by the mother,) contracted rheumatism some months since from exposure to cold and wet, while acting as baggage porter on the C. & A. Railroad line. All his articulations were successively attacked, the disease finally fixing on the left ancle, where it assumed a scrofulous character terminating in abcess and caries of the internal malleolus. The pain experienced was at first acute, mostly nocturnal, and evinced an unusual tendency to metastasis.

With the exception of the local affection as above, occasional aggravation of the rheumatic pains and a certain loss of power in the lower extremities, his general health continued unaffected.

He occasionally suffered from urinary irritation, but the attacks were of short duration, subsiding without treatment.

When first seen by me on the 23d of January, 1853, he had been confined to his bed for about two months from inability to rest his weight on the diseased ancle joint. He was of spare habit, but had not emaciated, his head long and gourd shaped was thrown backwards, and the knees strongly flexed. This position his mother had observed him to assume habitually, for some time previous. Skin dry and harsh; on the left internal malleolus was an ulcer communicating by a sinus wiih the joint.

The expression of his face was peculiarly sly and cunning, and his eye bright and restless. His intelligence was average. He complained principally of slight headache, and the rheumatic pain in the articulations. There was tenderness on pressure over the 2nd and 3d dorsal vertebræ, with loss of power in the lower limbs. Though his tongue was foul and his breath fetid, digestion was unimpaired, and his appetite always good, sometimes voracious. Pulse soft and natural, about 80 per minute. The heart and lungs were apparently healthy.

He was placed on the use of iodide of potassium, in five grain doses three times a day; a blister applied over the point of spinal tenderness. Dover's powder at bed time, with a stimulating liniment, was also ordered.

A day or two afterwards the urine was examined. It was

Magnified 120 diameters.

high colored, copious, acid, of sp. gr. 1015, and deposited an abundance of crystal of the oxalate of lime, with considerable urate of ammonia. The octahedra of the former salt were many of them enormously large, measuring from the 1-1000th to the 1-6008th of an inch. For this symptom the wine of colchicum in combination with liq. potassæ, was prescribed.

This treatment was continued for a few days without any material alleviation in the symptoms. Uric acid appeared in large amount in the urine; the oxalate of lime decreased in quantity, and the octahedra at the same time underwent a peculiar modification of form, being converted into dodecahedral crystals, by the bevelling off of their edges, as seen in the drawing on the preceding page, which also shows two varieties of uric acid found. The wine of colchicum and potassa were temporarily suspended, with a view of ascertaining their agency in effecting the above change; and in a few days the crystals resumed their original octohedral shape.*

On the 6th of February, after a comfortable night, the patient was suddenly seized with a dull, gravitative pain in the head, followed by vomiting and dimness of vision, and strong spasms principally affecting the posterior cervical muscles. His tongue was loaded with a thick, creamy fur, and he had not been to stool that morning. After a free purgation the attack subsided, leaving the patient, however, with slight headache and diminished sight of the right eye.

From this date, up to March 1st, he improved slightly: the ulcer seemed inclined to close under the lime-water dressing, and the rheumatic pains were not so severe. He still complained of headache; the spasmodic retraction of the head, together with the drawing up of the knees, continuing constant symptoms. (About

* The writer has observed the effect of the wine of colchicum in causing this modified form of crystal, which would seem to be a variety of oxalate passing into uric acid, in one other case, where the oxalic deposit also manifested a strong disposition to change into uric acid under its use. In that case, as in this, the subject was a negro. The urine of both contained unusually large crystals of the oxalate of lime, and both suffered from scro fulous disease, ending in the formation of abscesses. He is also led to believe, as the result of his observations in Dispensary practice, that gigantic specimens of the oxalate of lime are common in the urine of negroes affected with scrofula and phthisis, and are certainly far more frequently met with than in the white.

the second week in February the Ol. Morrhuæ was substituted for the iodide of potassium.)

On the 1st of March the headache, stiffness and spasm of the cervical muscles suddenly became aggravated; the pains being most severely felt in the occiput, thence shooting to the forehead.

Fever set in with a dry, hot skin, and a quick, exceedingly irritable pulse. The patient was very captious and unreasonable, manifesting a hastiness of temper, and suspicious watchfulness of those around him, altogether foreign to his naturally amiable disposition. He complained of intolerance of light. His appetite continued good, and his bowels regular; the urine was heavily loaded with urate of ammonia.

Gradually the pains increased in violence, extending to the muscles of the back and loins, and on the fourth, after a sudden aggravation of all the symptoms, they became paroxysmal, occurring at intervals of half an hour with such extreme severity as to cause the patient to scream with agony. During these paroxysms, spasms of the posterior cervical muscles and of the extensors of the back, analogous to those occurring in tetanus, were observed. The head was violently retracted, often with extreme suddenness at the onset of the attack; the pupils were dilated and sluggish, and vision dim and imperfect. The fever also increased, and there was not unfrequently perversion of the mental faculties with delirium.

During the interval the patient was rational, though inclined to drowsiness, and the fever declined: but there was only a partial relaxation of the tonic spasm. The Ol. Morrhuæ was now suspended; cups were freely applied to the back and loins; the sore ancle freshened up by a blister, and calomel, in combination with Dover's powder, given in moderate doses, four or five times daily.

Under this treatment he again improved, and the urgency of his symptoms had somewhat abated, when on the seventh (three days afterwards) he was attacked by the most agonizing cramps in the abdominal muscles, which rapidly invaded the lower extremities. For the relief of these, sinapisms were freely applied, and after the usual remedies had been exhausted, without any effect, inhalation of ether was cautiously tried. This agent proved successful, the cramps disappeared, and he slept for some hours.

Almost immediately on waking he had a short chill to which succeeded violent fever, headache, and a return of the spasm in the head and neck with augmented violence. Up to the twelfth (March) he suffered constantly, finding no relief night or day.

At this time the disease assumed a periodic character, with a daily exacerbation, beginning about noon and lasting till late in the evening. The following note of his condition was made on the 12th, during the attack:

"Found the patient suffering the most excruciating pain, principally referred to the occiput, and back of the neck. Flexion of the head backwards is so violent that the occiput nearly touches the spine. He is entirely blind, rolling his eyes wildly around, at times fixing them with an amaurotic stare: pupils are dilated, very sluggish: hearing appears morbidly acute, the least noise causing him great distress. When his attention is strongly fixed, he answers questions rationally, but when not roused his mind wanders and he talks incoherently. The pulse is about 125, quick and irritable, and he has considerable fever: appetite is excellent and bowels open."

Towards midnight the remission usually occurred: the spasm relaxed in a measure, sight returned, the fever and headache declined, and he would obtain a few hours of quiet sleep.

Sulphate of quinia, at first in moderate and subsequently in large doses was administered, combined with Dover's powder, calomel and aloes; counter-irritation was kept up upon the spine and extremities. Marked benefit seemed for a short time to follow this treatment, the paroxysms occurring with mitigated severity an hour or two later every day; but on the 20th of the same month, he was again attacked with cramp over the entire body, ending as before in a concentration of the spasm in the head and neck. The left eye became entirely amaurotic, and shortly after the sight began to fail. The retraction of the head, and rigidity of the cervical muscles, increased to an almost incredible degree, the occipital pain (especially at night) being so insupportable to the patient that his screams were incessant, alarming the entire neighborhood in which he lived. In his agony he would seize his head with both hands, and frantically endeavor to raise it from the pillow; failing in this way to obtain relief, he would force his mother to sit for hours by his bedside making firm pressure against his temples with a knotted handkerchief. The pulse about this period averaged 125 to 130 beats per minute.

No material change in the symptoms was noted up to the 28th of March, on the morning of which day the patient had a strong convulsion lasting about an hour, affecting the muscles of the trunk and upper extremities. He remained in a stupid, semi-comatose condition until early next morning, when another and less violent convulsion followed, on emerging from which his right arm was observed to be partially paralysed, and there was ptosis of the left eyelid. Intellect in a measure returned, but ever after the influence of the brain over the voluntary muscles was imperfectly and irregularly exercised, nor could the patient execute even the simplest movement without assistance. Violent spasmodic twitchings of the muscles, both voluntary and involuntary, constantly harassed him sleeping and waking; these were often as irregular and unaccountable as the convulsive movements in a bad case of chorea, being usually most frequent and severe at night.

Amaurosis of the remaining eye speedily followed, the pupils of both were dilated to a very considerable size, and the sphincter ani often so rigid and wirelike that the nozzle of an ordinary injection pipe could with great difficulty be introduced. About this time the penis was frequently observed to be in a state of semi-erection, and whenever this happened, dysuria was sure to follow. (The urine at this date contained abundance of urate of ammonia and hippuric acid, the oxalate of lime having nearly disappeared. On standing a short time phosphates were rapidly deposited.)

Gradually from this period up to the time of his death, with the exception of one or two lucid intervals of short duration, the intellectual faculties all seemed to become merged in the animal. The patient ceased to complain of pain, or only vaguely alluded to its existence: nor when questioned could he indicate where he suffered. Digestion languished, and the bowels, previously soluble, required the frequent administration of drastic purges. Yet his appetite continued ravenous, and unless frequently supplied with food he would constantly and querulously call for it, devouring large quantities mechanically, with a peculiar lateral grinding movement of the lower jaw, similar to that of a ruminating animal, at the same time smacking his lips with an apparent air of intense satisfaction. The act of mastication continued for some time after the bolus of food had been swallowed. Deglutition seemed painful and difficult, a fact readily accounted for by the unnatural flexion of the neck. For the last few days of his life, the treatment amounted to almost nothing. He was allowed a generous diet, and counter-irritation kept up on the spine and extremities. On the 18th of April the pulse became more feeble and very rapid; dysphagia increased to such an extent that no food, solid or liquid, could be taken; rigid spasm of the sphincter ani, with obstinate constipation, supervened, and the patient expired in a convulsion on the afternoon of the 23d.

An autopsy was made by me 38 hours after death, at which Dr. J. J. Levick was present, and assisted; rigidity of the cervical muscles and retraction of the head noticed, though decomposition had commenced.

The *head* only was examined. On the removal of the calvaria, considerable injection of the dura mater was found, with unusual development of the Pacchionian glands: the sinuses were gorged with blood. The dura mater being opened, the other membranes were found healthy and altogether free from traces of inflammatory action.

The convolutions on the superior aspect of the brain presented nothing abnormal. The anterior lobes were now cautiously raised, and the olfactory and optic nerves exposed. The former appeared healthy, the latter were soft and somewhat flattened. They were next divided, and immediately behind the chiasm of the optic nerve adhesions were found to exist between the dura mater and the brain. At this point, a small hydatid cyst was discovered bound down by the adhesions, and a few granular patches of lymph in the right fissure of Silvius.

The tentorium was then divided, and the cerebellum carefully raised. In doing this a considerable quantity of thick greenish pus escaped, and the remains of an encysted abscess, about the size of a large walnut, occupying the floor of the right lobe of the cerebellum, exposed.

In the superior portion of the cerebellum, about the middle line, (processus vermiformis,) was found another abscess equally large, and also encysted. This was dissected out entire, which could be readily done owing to the toughness of the sac, and its contents discovered to be in all respects similar to those of the first. Six other cysts, (making 8 in all,) of smaller size, occupied various parts of the cerebellum, which organ was elsewhere much broken down and softened, presenting a honey-combed appearance.

The *cerebrum* was in all respects healthy, and although a minute and careful examination was made, no traces of softening or inflammatory action could be detected. The ventricles contained a little serum.

The medulla oblongata as far as removed exhibited no signs of morbid alteration.

Attention was next directed to the base of the skull, more especially to those parts about the optic tract. The pituitary gland, enlarged to about four times the usual size, was bound down by numerous adhesions to the sella turcica, and on removing it the subjacent bone was found to be carious. On making pressure pus oozed out. This softened condition of the bone extended to a point beneath the chiasm of the optic nerves, which were, as before remarked, flattened and softer than in health. No other pathological lesion was noticed.

In hastily reviewing the leading points in the above case, the following seem more especially worthy of attention.

*First.* The insidious approaches of the disease, indicated for weeks prior to the appearance of its recognized symptoms by slight tonic spasm of the cervical muscles, with a similar condition of the flexors of the lower extremities. *Second.* Its rheumatic origin, subsequently modified by the scrofulous habit of the patient and the disposition to sudden metastasis. *Third.* Its localization in and final destruction of the cerebellum without any notable lesion of the cerebrum. *Fourth.* The diseased condition of the pituitary gland, and its apparent connection with the caries of the subjacent bone, the softening of the optic nerves, and resulting amaurosis. *Fifth.* The color of the patient in its bearings on the urinary deposits.

The symptoms developed during the progress of the disease: the obstinate tonic spasm: the violent and increasing pain in the occiput: the absence of paralysis even partial up to a comparatively late period: the frequent pulse; the inordinate appetite: rarity of vomiting as a symptom: the usually soluble condition of the bowels, and the frequent semi-erections of the penis, point to a lesion of the cerebellum. At the same time it is not to be denied that symptoms of an essentially cerebral character were present, such as the gradual decay and final obliteration of the mental faculties, the convulsions, &c., and these the absence of all tangible local lesion of the cerebrum, renders it difficult to explain. The doctrine of sympathetic irritation would seem to be insufficient to account for the persistence and gravity of these symptoms, and the location of the abscesses forbids the idea that pressure could have been the cause; perhaps the most satisfactory explanation is afforded by supposing a molecular alteration in the cerebral substance.

## Figures and Tables

**Figure f1:**